# Supplementary material for: Nitric Oxide Pre-Treatment Advances Bulblet Dormancy Release by Mediating Metabolic Changes in Lilium
Source: Int J Mol Sci. 2024 Dec 27;26(1):156. doi: 10.3390/ijms26010156 (PMC11720527; doi:10.3390/ijms26010156)
Supplement: Supplementary file 1 [file ijms-26-00156-s001.zip › ijms-3362233-supplementary.pdf]

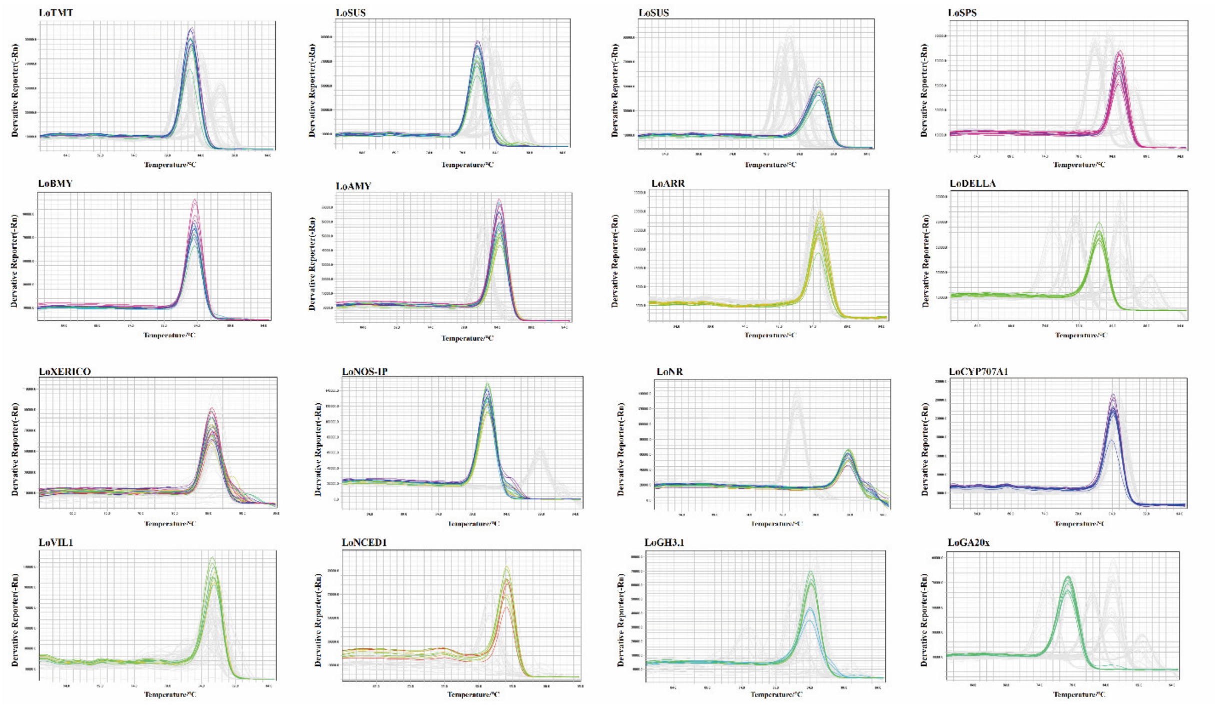

Figure S1. Melting curves of each gene obtained during qRT-PCR analysis.

Table S1. Details of the concentrations of SNP and c-PTIO used in the preliminary experiment.

Sample	Bulb length(cm)	Decay ratio (%)
CK	4.34±0.71	0
0.5 mM SNP	4.38±0.45	0
1 mM SNP	4.39±0.26	0
2 mM SNP	4.29±0.84	0
5 mM SNP	6.13±0.64	0
10 mM SNP	7.60±0.57	0
20 mM SNP	6.47±0.46	0
30 mM SNP	6.25±0.77	23.3
0.5 mM c-PTIO	4.21±0.82	0
1 mM c-PTIO	2.11±0.23	0
2 mM c-PTIO	2.35±0.63	20
5 mM c-PTIO	2.46±0.87	56.6
